# Blind to Bias: The Impact of Prescriptive Age Norms on Perceived Age Discrimination

**DOI:** 10.1093/geroni/igaf122.916

**Published:** 2025-12-31

**Authors:** M Clara de Paula Couto, Klaus Rothermund

**Affiliations:** Friedrich-Schiller-University Jena, Jena, Thuringen, Germany; Friedrich-Schiller University Jena, Institute of Psychology, Jena, Thuringen, Germany

## Abstract

Age discrimination is prevalent in many societies, impacting psychological well-being, self-esteem, and health. It can be experienced personally (directly) or socially (by observing others being targeted). A key factor shaping these experiences is the presence of prescriptive age norms. For example, when older adults are expected to withdraw from social roles to make way for younger generations (the disengagement norm), violating these expectations may lead to age discrimination and feelings of guilt or shame. This study investigates whether the endorsement of prescriptive age norms influences the perception of age discrimination across age groups. Strong adherence to these norms may cause older individuals to not perceive or trivialize age discrimination and to internalize blame for their experiences instead of attributing it to external factors. The sample was drawn from the Aging-as-Future questionnaire study, which included 2,902 participants aged 40 to 90 years. Moderated regression analyses examined the relationship between endorsement of disengagement norms and perceived age discrimination, with age as a moderator. Results showed that stronger endorsement of disengagement norms was linked to fewer reports of age discrimination, particularly among older adults. In conclusion, this study highlights the complex role of prescriptive age norms, suggesting that strong endorsement of disengagement norms may reduce sensitivity for age discrimination. Understanding these norms’ psychological impact is crucial for addressing age discrimination across different age groups.

